# Recycled fibers from pre- and post-consumer textile waste as blend constituents in manufacturing 100% cotton yarns in ring spinning: A sustainable and eco-friendly approach

**DOI:** 10.1016/j.heliyon.2022.e11275

**Published:** 2022-10-26

**Authors:** Yeasin Arafat, Ahmed Jalal Uddin

**Affiliations:** a Quality Assurance Department, PHP Spinning Mills Limited, Hathazari Road, Natun Para, Chittagong, Bangladesh; b Department of Yarn Engineering, Bangladesh University of Textiles, Tejgaon, Dhaka 1208, Bangladesh

**Keywords:** Pre- and post-consumer textile waste, Recycled fiber, Ring spinning, Mechanical shredding, Sustainable and eco-friendly approach

## Abstract

The usage of recycled fibers has achieved enormous importance in the textile sector due to growing environmental awareness, legal requirements for more sustainability and the cost of raw materials. Recycled cotton fibers derived from mechanical shredding of textile waste possess lower quality values and therefore they are spun through blending with other fibers in rotor spinning system for the production of coarse yarns (10–20 Ne) to make denim, towel and home furnishing. Owing to low fiber migration, rotor yarns require high twist during spinning which makes them stiffer and poorly moisture absorbent. Rotor yarns also feel harsh in skin contact due to the presence of wrapper fibers in the yarn surface. Intending to make knit top garments like T-shirts and polo shirts, apparel manufacturers nowadays demand recycled fiber-contained soft and high moisture absorbent yarn that can be produced in ring spinning system. In the current endeavor, cotton fibers reclaimed from pre- and post-consumer textile waste were blended with virgin cotton and soft-twisted 30 Ne yarns were manufactured in ring spinning frame on an industrial scale in a spinning mill. A thorough analysis of the structure and properties of the yarns revealed that up to 25% recycled cotton fibers can be used as an alternative to virgin cotton to manufacture medium count (30 Ne) yarn in ring spinning suitable to produce knit top garments.

## Graphical abstract

**Figure:**
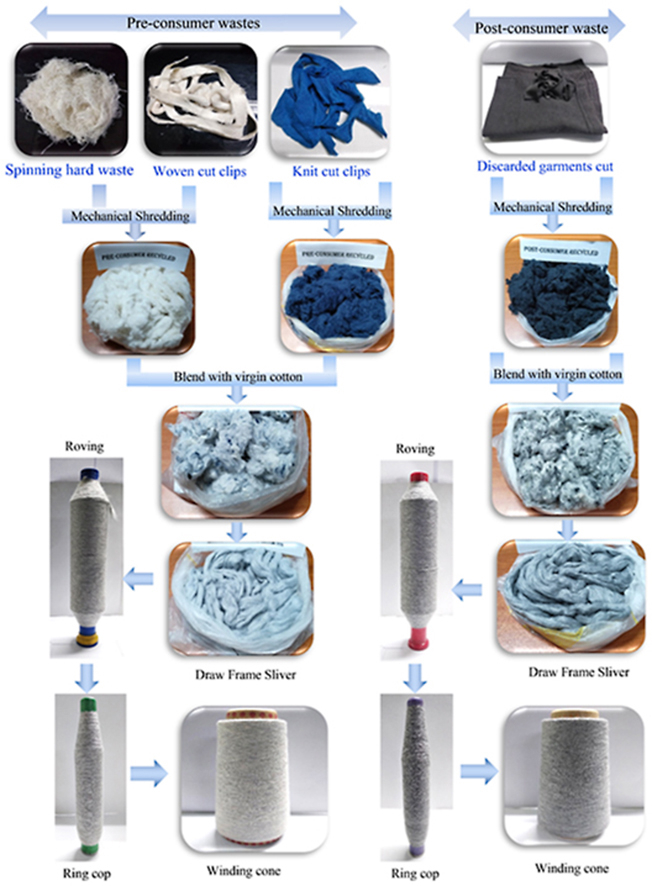

## 1 Introduction

Cotton is all-natural, and its fabric is soft, absorbent, comfortable, highly breathable and hypoallergenic (Kılıç et al., 2020). Therefore, consumers have a high opinion of all cotton and cotton-rich apparel. Cotton accounts for 25% of all fibers used in textiles (Garside, 2020). Its consumption is consistently on the rise due to the increasing demand of the world's population and mitigating impact of COVID-19 in especially cotton consumer countries like China, Bangladesh, Pakistan, India and Mexico. Worldwide, cotton prices have also been rising since July 2021 due to global demand (Global cotton production & consumption to improve: TexPro, 2021).

Cotton cultivation is vital for mankind but it has severe environmental impacts. Run-off from cotton fields contains harmful pesticides and fertilizers those pollute water systems like rivers, lakes, marine beaches, and also underground aquifers (Ütebay et al., 2019). Other than cotton cultivation, the textile industry is a large source of pollution and a waste-generating sector in the world (Stanescu, 2021). Textile waste is produced in every phase of the textile manufacturing process, viz. spinning, weaving, knitting, dyeing, finishing, garment manufacturing and even at the consumer end (Jamshaid et al., 2021). In addition, with rapid growth and evolution in fashion trends along with the fast throwaway culture of the new generation, textile production and waste generation rates have increased substantially over the last decades (Ütebay et al., 2019; Sun et al., 2021; Rahman et al., 2022). Globally, around 87% of total discarded textiles, of which around 90% are reusable and recyclable, ended up through landfill or incineration creating a serious environmental threat (Moazzem et al., 2021).

Aiming to protect the environment from pollution and waste, Environment Protection Act 2017 formulated a framework for how to comply with the new waste duties that are applied to people who generate, transport or receive industrial waste (Summary of Waste Regulations, 2022). The act supports and encourages the recycling of waste and resource recovery to divert as much waste from landfill as possible. The Global Recycled Standard (GRS) is an international standard for tracking and verifying the content of recycled materials in a final product complying with environmental regulations. GRS covers the manufacturing of products containing at least 20% recycled material. The GRS applies to the companies involved in the ginning, spinning, weaving, knitting, dyeing, printing, and sewing processes in more than 50 countries (Global Recycled Standard, 2011).

Textile waste can broadly be classified as soft waste and hard waste. Soft wastes generated from the blow room, carding and combing section of the spinning mill are reused in rotor spinning to produce coarser yarns mainly for denim. Hard wastes are of two types: pre-consumer and post-consumer waste (Sandin and Peters, 2018). Pre-consumer wastes are derived before they reach the consumer. They are generally clean waste and can be either yarn or fabric and need recycling to be put again in production. Conversely, post-consumer wastes are mostly garments that are discarded after use. They can also be recycled to use again in the manufacturing process to produce similar or other products (Ajila, 2019).

In the context of facing a high demand for cotton and the environmental regulations on the waste management system, the development of a sustainable strategy to recover cotton from textile waste is clear in an eco-responsible approach (Yousef et al., 2019). As the cost of cotton fiber accounts for the majority (over 50%) of the production cost of ring-spun yarn (Oner et al., 2019), the reduction of raw material costs by utilizing recycled cotton will provide considerable advantages to the spinners and consumers (Parsi et al., 2016). Recycling can be accomplished by extracting fibers from waste yarns and fabrics using a mechanical shredding machine that vigorously strikes and tears them into their original fiber components. However, recovered fibers obtained in this way manifest very short lengths and small lumps of fibers. That's why these fibers solely cannot be converted into yarns by spinning. To get rid of this problem, virgin fibers are blended with recycled fibers those act as carriers during the spinning process, and using this technique, coarser yarns were produced in rotor spinning system (Ute et al., 2019; Yuksekkaya et al., 2016). For instance, 10–20 Ne rotor yarns were produced from virgin cotton and recycled fibers derived from garment wastes with different blend ratios (cotton: recycled fibers 10:90 to 75:25) (Memon et al., 2022; Kanan et al., 2022; Awgichew et al., 2021; Jamshaid et al., 2021; Ute et al., 2019). Rotor spinning is limited to producing only coarser yarns (theoretically up to 24 Ne, commercially up to 20 Ne). Due to poor fiber migration, rotor yarns necessitate higher twists to get similar strength to ring-spun yarn which makes them stiffer and low moisture absorbent. In addition, rotor yarns hold a high number of disoriented folded fibers, called ‘wrapper fibers’ or ‘belts’. Wrapper fibers are not completely tied into the yarn body. They have free ends that wrap themselves around the yarn periphery causing constriction of the yarns making the fabric harsh (Lawrence, 2010). This limits the usage of rotor yarns for a narrow range of applications such as denim, towel and home furnishing (Ernst, 2014).

On the contrary, ring spinning is deemed to be the most widely used spinning system because of its versatility in producing low-twisted soft, absorbent yarn (for knitting) to moderate and hard twisted yarns (for weaving and crepe yarn), very coarse (2 Ne) to super fine yarns (200 Ne), custom yarn to functional yarns, etc. (Shaikh and Bhattacharya, 2016; Chattopadhyay and Sinha, 2007). Any type of fiber such as natural (cotton, flax, wool etc.), synthetics and their blends can be spun in this system efficiently. Moreover, ring spinning occupies a unique position among the various commercial spinning systems, setting the benchmark for the highest yarn strength. The ‘tension migration’ in a spinning triangle is unique in ring spinning that produces a high degree of intermingling of the fibers in the yarn cross-section ensuring better translation of fiber strength into yarn strength (Lawrence, 2010). Since ring yarns do not possess wrapper fibers in their structure, thereby present a smooth feeling to the wearer. Some fabric properties produced from rotor and ring yarns are compared in Table 1 (Gedilu et al., 2022; Jhanji, Y et al., 2021). However, due to the above-mentioned reasons, ring yarns are preferred to produce knit top garments such as T-shirts and polo shirts.

**Table 1:** Properties of knit fabrics produced from rotor and ring yarns.

Properties	Rotor yarn	Ring yarn
Moisture vapor transmission rate (g/m^2^/24 h)	6.31–8.81	7.25–9.46
Water vapor permeability (kg/m^2^ s Pa)	0.142	0.131
Thermal insulation (Km^2^/W)	4.54	4.88
Low-stress compression resilience (%)	7.71	4.47

Apparel manufacturers nowadays want sustainable and eco-friendly yarns containing recycled fibers to produce knit top garments like T-shirts and polo shirts. In this case, improved wearability such as a smooth and soft feel with high moisture absorbency of yarns is desirable. Such yarns can be produced in ring spinning system with lower twists with careful controlling of balloon tension due to the existence of recycled short fibers. Utilizing recycled cotton fibers obtained from pre- and post-consumer yarn and fabric wastes, the current attempt was undertaken to manufacture medium-count yarn in ring spinning frame with a lower twist suitable to produce knit top garments useful for all users irrespective of age and gender.

## 2 Materials and methods

**Figure 1:**
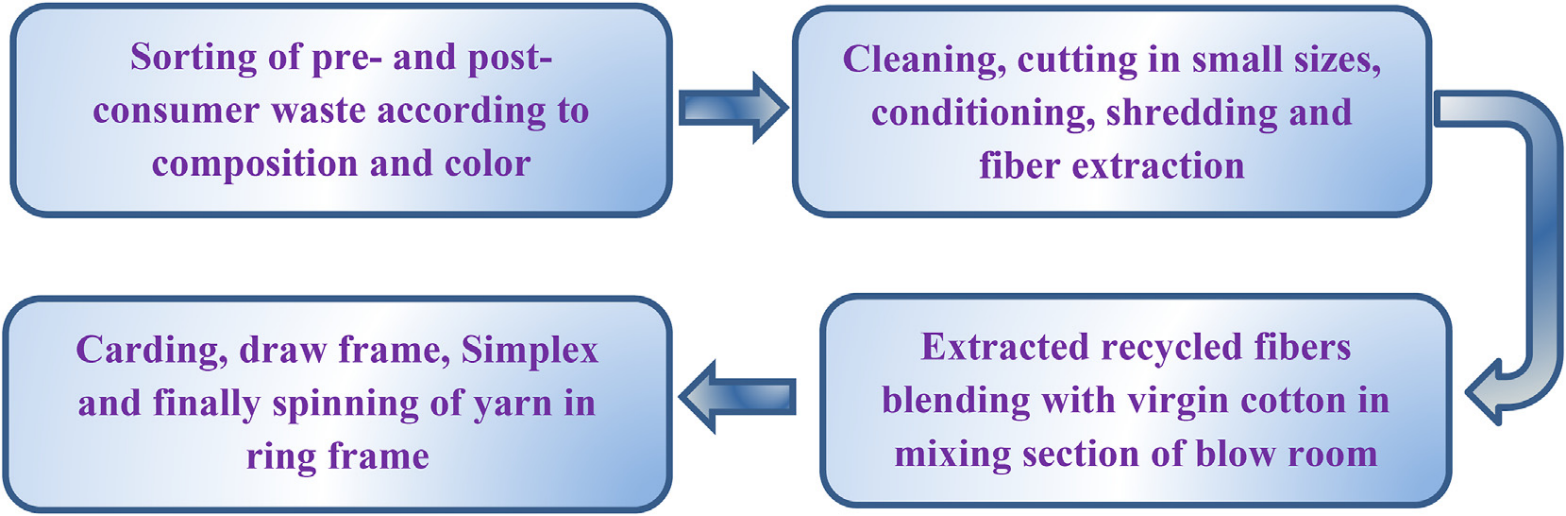
Flow chart of manufacturing ring-spun yarns from blends of virgin cotton and recycled cotton fibers extracted from pre- and post-consumer textile wasts.

Blend yarns from pre- and post-consumer recycled cotton and virgin cotton were manufactured in ring spinning line following the steps given in Figure 1.

### 2.1 Material preparation

Mechanical recycling of textiles is the process of recycling the textile fabric back into fibers without using any chemicals. In the current work, textile pre- & post-consumer wastes were mechanically processed into recycled cotton in accordance with the following steps.

#### 2.1.1 Material sorting

**Figure 2:**
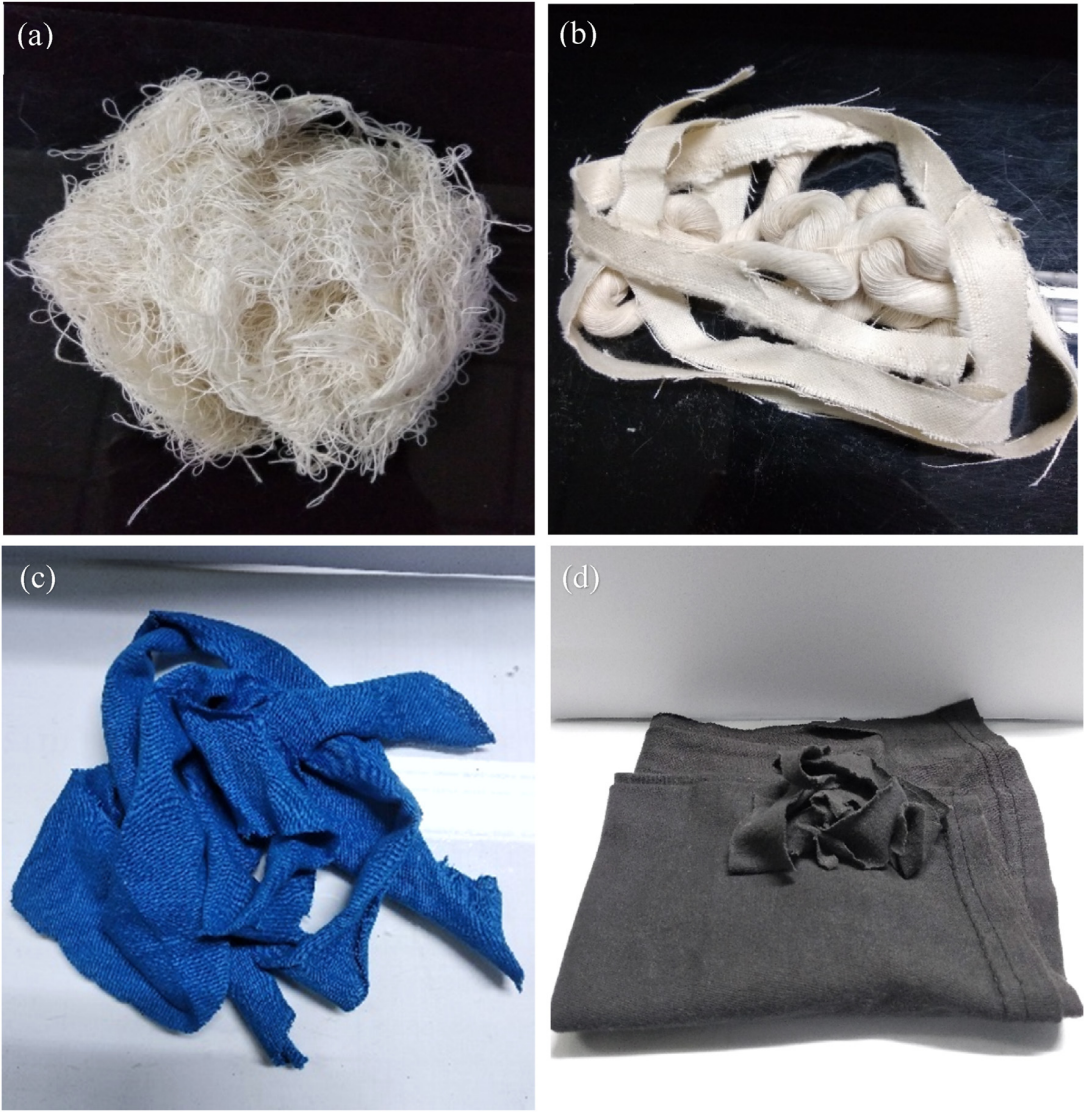
Pre-consumer textile wastes: (a) spinning hard waste**,** (b) woven cut clips and (c) knit cut clips. Post-consumer textile waste: (d) garments cut clips procured from second-hand clothing shop.

The fabric waste is first sorted on the basis of fiber composition and color. For this work, 100% cotton waste, namely spinning hard waste (derived from the splicing operation of winding section of spinning mills), weaving waste (generated after the production of fabric in the weaving mill) and navy-blue color single jersey knit cutting clip (found available in garments of Bangladesh) were selected as pre-consumer waste (Figure 2a, b and c). The blend ratio was 50% hard waste: 25% woven cut clips: 25% knit cut clips. Black color garments (procured from second-hand apparel shop) was used as post-consumer waste (Figure 2d).

**Figure 3:**
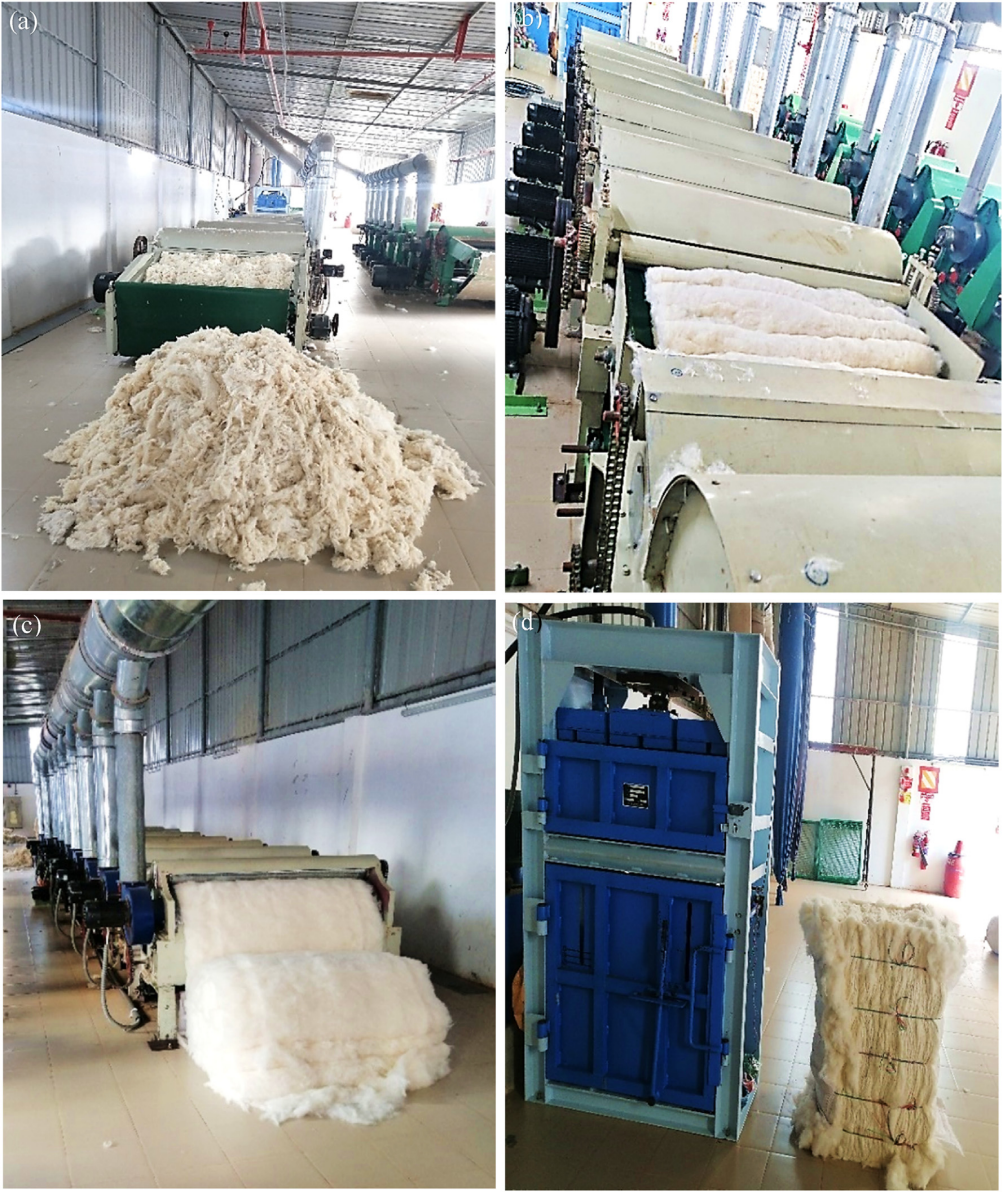
Mechanical recycling: (a) material feeding to shredding machine, (b) shredding in several zones, (c) material delivery after shredding, and (d) final bale making.

**Figure 4:**
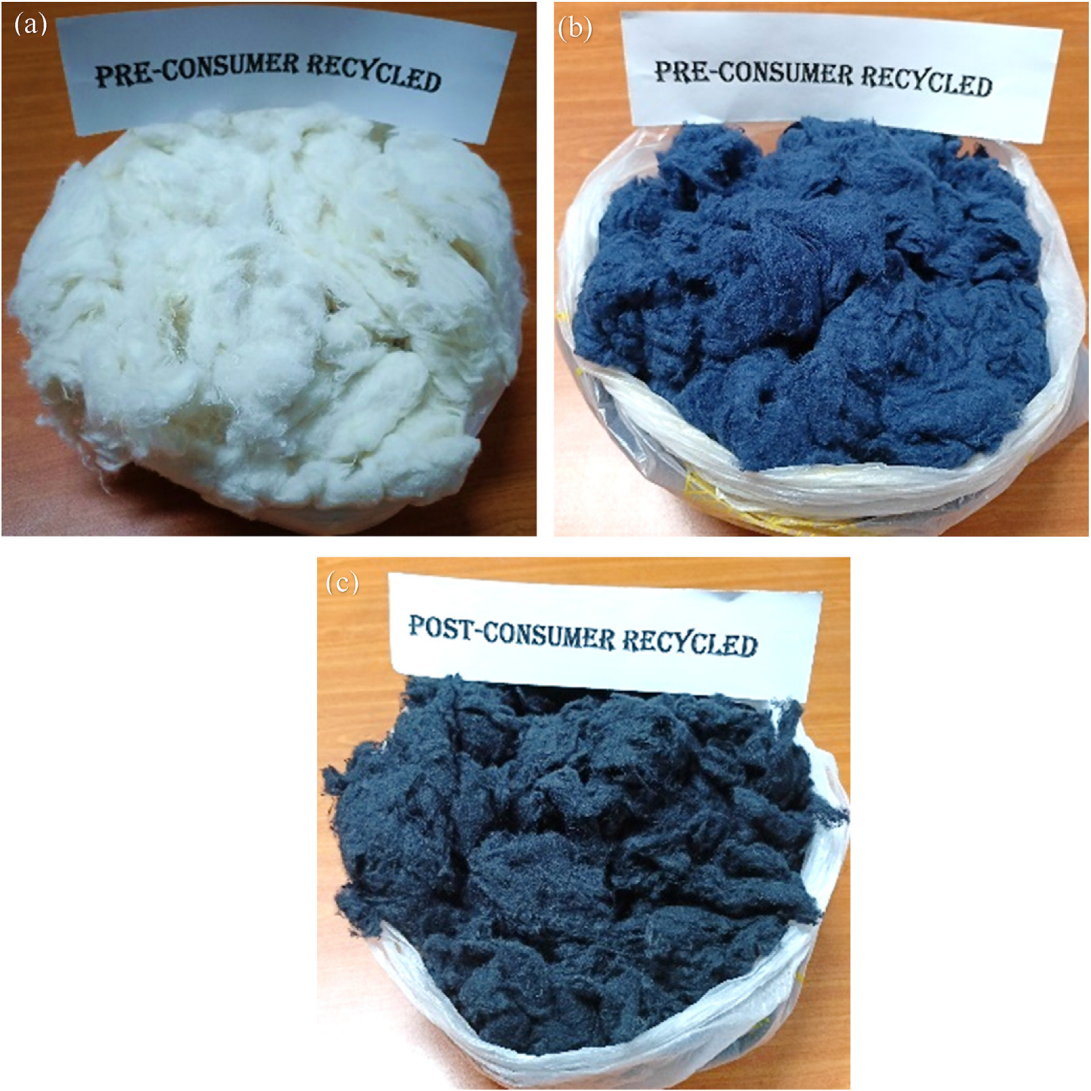
After shredding, pre-consumer recycled fiber obtained from (a) spinning hard waste & woven cut clip, and (b) knit cut clip. Post-consumer recycled fibers obtained from (c) second-hand garments cut clips.

After sorting, the waste materials were cut into small pieces by passing through a cutter. After that, they were conditioned (by spraying 2% water on the weight of the fabric) for at least 12 h to avoid any fire incidence during shredding. Figure 3(a), (b) and (c) show the images of the shredding machine. The machine consists of six carding cylinders clothed with opposite sets of strong sharp wires those tears the yarns and cut pieces of fabrics. Finally they are converted into component fibers by exerting heavy and rough carding actions made by sharp wires of carding cylinders. Extracted fibers thus obtained are made into bales (Figure 3d) that are later shifted to the mixing room. Pre- and post-consumer recycled fibers obtained after shredding are shown in Figure 4a, b and c.

### 2.2 Properties of recycled fibers

Even after being shredded and opened vigorously in a shredding machine, recycled fibers still had many hard lumps of fibers. Hence, characterization of recycled fibers was not possible in Uster High Volume Instruments (HVI) since the Program Logistic Control (PLC) software of HVI was basically designed to test only fully opened and grey-color cotton fibers. An instrumental jam with an error signal was observed while attempting to test recycled color fibers in HVI. However, recycled fibers were finally characterized in Uster Advanced Fiber Information system, AFIS Pro-2 according to ASTM D5866-05 method. Test results of virgin cottong and shredded fibers are shown in Table 2. In the blow room and carding, a good amount of trash and short fibers are removed as dropping, taker-in waste and flat waste. Some neps are formed in the blow room during opening and beating action, and some are removed in carding. Hence, the fiber parameters were studied again in finisher draw frame slivers which are exhibited in Table 3.

**Table 2:** **S**pecifications of virgin cotton, and pre- and post-consumer recycled cotton.

Test Parameter	Virgin cotton	Pre-consumer recycled cotton (Spinning hard waste + woven cut clips + knit cut clips)	Post-consumer recycled cotton
Total Neps Count [Cnt/g]	217	463	523
Total Neps mean size [μm]	676	728	885
Upper Quartile length, UQL (w) [mm]	28.3	22.2	18.9
Short fiber content, SFC (w) % [<12.7 mm]	10	35	45
Fineness [mtex]	158	164	168

**Table 3:** **S**pecifications of virgin cotton, and pre- and post-consumer recycled cotton in finisher slivers.

Test parameter	Virgin Sliver	Pre-consumer recycled sliver				Post-consumer recycled sliver			
		10%	20%	25%	30%	10%	20%	25%	30%
Total Neps Cnt [Cnt/g]	89	113	145	155	175	122	160	168	192
Total Neps mean size [μm]	612	645	658	670	689	688	697	712	728
UQL (w) [mm]	28.8	28.5	28.3	28.1	27.9	28.2	28.0	27.9	27.7
SFC (w) % <12.7 mm	8.0	9.1	11.2	12.3	13.4	10.4	14.0	15.7	17.5
Fineness [mtex]	152	154	155	157	158	157	160	162	163

### 2.3 Methodology

At first, pre-consumer recycled cotton was blended with virgin cotton with four different blend ratios (10:90, 20:80, 25:75 and 30:70). Similarly, post-consumer recycled cotton was also blended with virgin cotton with the same blend ratios. All blends were then converted into yarns in ring spinning frame. The spinning of blend yarn with a blend ratio 35:65 i.e. containing 35% recycled fibers was also possible in ring frame but produced yarn was too weak to sustain the stress during knitting and weaving. That's why the discussion of the yarn with that blend ratio was not included in this article.

**Figure 5:**
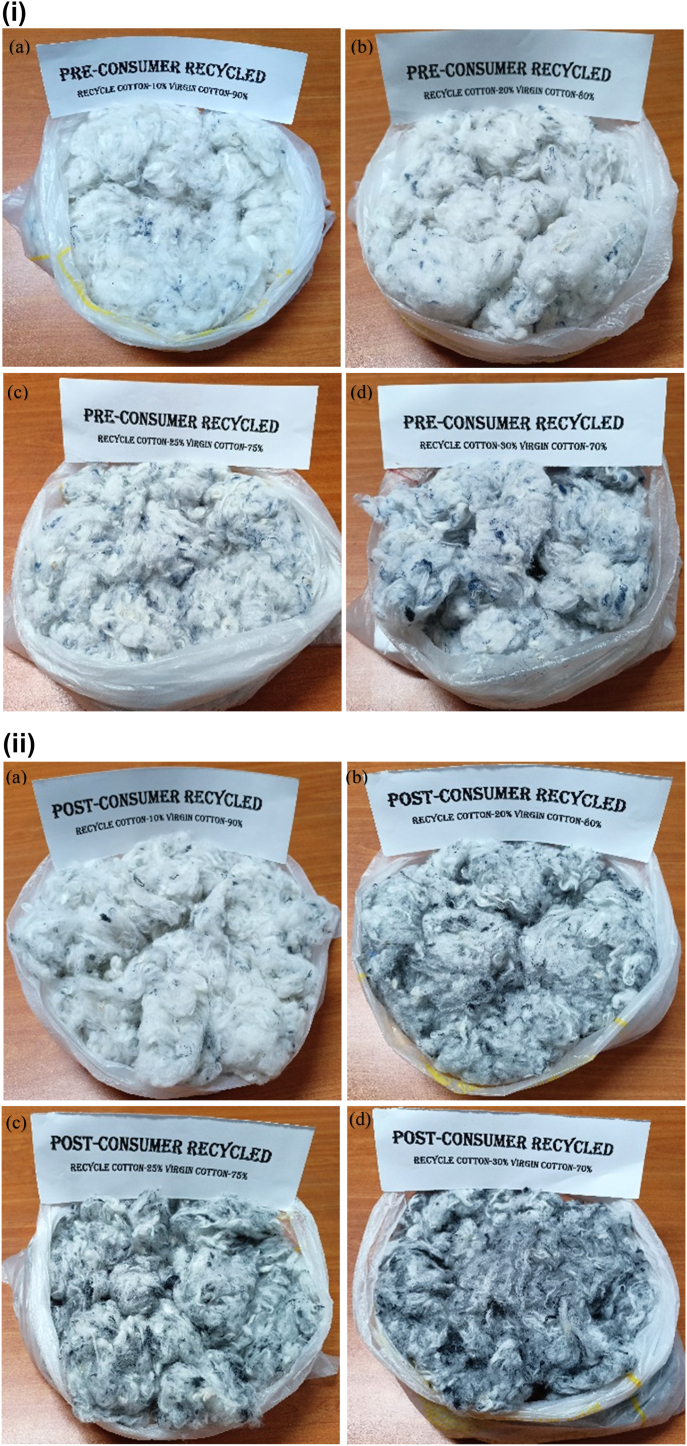
(i) Complete mixing of pre-consumer recycled cotton/virgin cotton with blend ratio (a) 10/90, (b) 20/80, (c) 25/75 and (d) 30/70. (ii) Complete mixing of post-consumer recycled cotton/virgin cotton with blend ratio (a) 10/90, (b) 20/80, (c) 25/75 and (d) 30/70.

At first, recycled cotton and virgin cotton fibers were placed in one place. The required amount of fibers was then taken for blending according to the blend ratio after weighing in an electric balance. Then the fibers were mixed thoroughly by hand. The complete mixing of pre- and post-consumer recycled cotton and virgin cotton fibers with different blend ratios are displayed in Figure 5(i) and (ii) respectively.

### 2.4 Material feeding and preparation of drawn slivers

After completing the blending and mixing, materials were passed through a sample blow room line consisting of a feed lattice and fine cleaner. Materials were then fed to carding machine through chute feed. Carding is the second process of spinning after blow room which converts the feed materials into a uniform strand of fibers called sliver.

**Figure 6:**
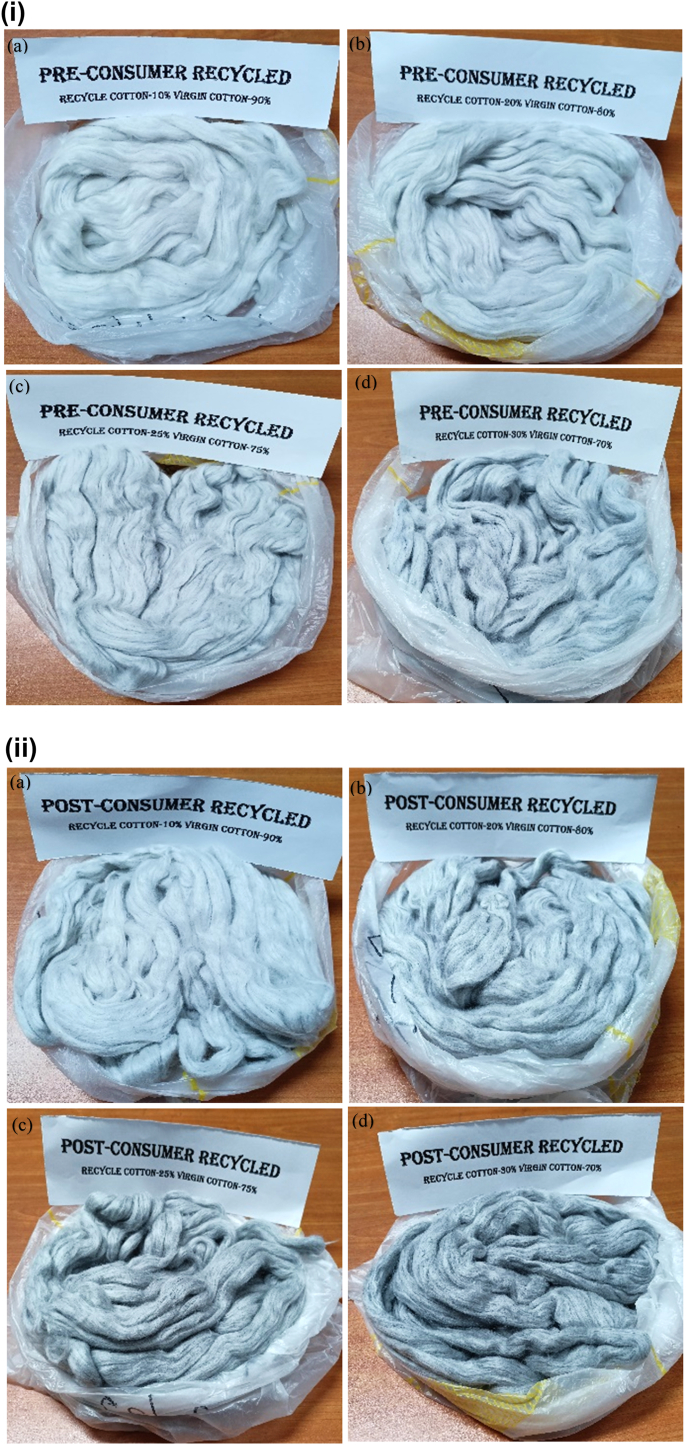
(i) Finisher draw frame sliver made with pre-consumer recycled/virgin cotton fiber with different blend ratio (a) 10:90, (b) 20:80, (c) 25:75 and (d) 30:70. (ii) Finisher draw frame sliver made with post-consumer recycled/virgin cotton fiber with different blend ratio (a) 10:90, (b) 20:80, (c) 25:75 and (d) 30:70.

The short- and medium-term variations of carded slivers are equalized by the doubling action in the draw frame. Moreover, hooked, curled and crimped fibers present in carded slivers are straightened and oriented by the drawing action of two passages of the draw frame machine, namely breaker draw frame and finisher draw frame. After two draw frame passages, intimate blending between recycled cotton and virgin cotton were achieved as shown by the sliver images in Figure 6(i) and (ii).

### 2.5 Manufacturing of roving and yarn

A total of eight roving samples, four for pre-consumer recycled cotton and four for post-consumer recycled cotton blended with virgin cotton with four blend ratios (10:90, 20:80, 25:75 and 30:70) were prepared.

The objective of this work was to produce textile wastes/virgin cotton blend yarns with a lower twist in ring frame suitable for top knit garments. By dint of heartiest effort along with strict process control, 30 Ne ring yarns incorporating up to a maximal 30% pre- and post-consumer waste with virgin cotton were possible to manufacture in an industrial scale ring spinning frame. Since pre- and post-consumer recycled cotton possess more short fibers (shown before in Table 2 and Table 3) compared to that of virgin cotton, careful controlling of balloon tension in ring spinning was very crucial to achieve a stable spinning condition without frequent end breakage.

**Figure 7:**
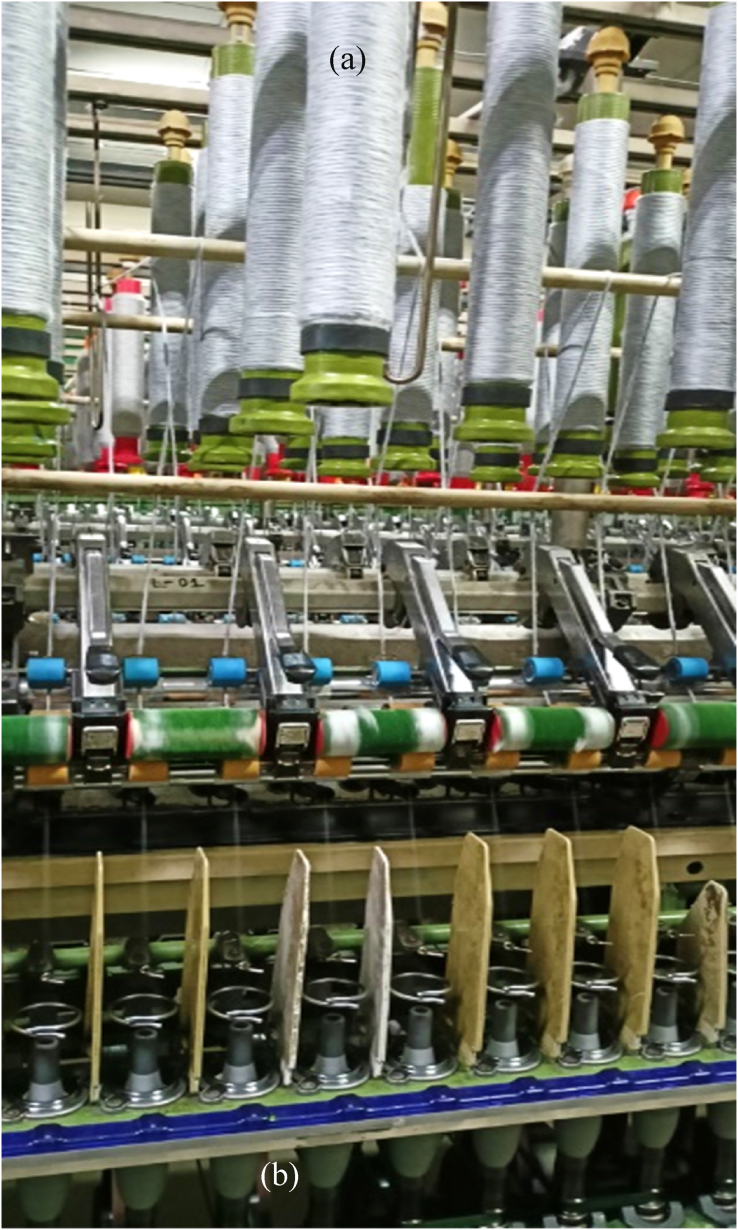
Manufacturing of recycled cotton and virgin cotton blend yarn in ring spinning frame: (a) roving and (b) ring cop.

For comparison purposes, a 30 Ne yarn sample with 100% virgin cotton was also prepared with the same spinning parameters. The detail of the machines and technical parameters to produce yarns from consumer wastes are given in Table 4. Figure 7 illustrates the manufacturing of recycled cotton/virgin cotton blend yarns (Figure 7b) from roving (Figure 7a) in ring frame.

**Table 4:** Production details for the preparation of roving from recycled cotton and virgin cotton.

Process	Machine & Model	Country of origin	Delivery Material	Production speed
Fiber Shredding	Balkan	Turkey	Reclaimed fibers	900 kg/h
Blow Room	Rieter	Switzerland	0.0012 Ne card mat	Chute feed to card
Carding	Rieter C-60	Switzerland	0.104 Ne carded sliver	60 kg/h
Breaker Draw Frame	Rieter S BD10	Switzerland	0.11 Ne drawn sliver	650 m/min (6 doubling)
Finisher Draw Frame	Rieter RS BD22	Switzerland	0.11 Ne drawn sliver	600 m/min (8 doubling)
Simplex	Honyuan 492	China	0.8 Ne roving (TPI 1.15)	1000 rpm
Ring Frame	Lakshmi LR-6	India	30 Ne yarn (TPI 21.9)	14,000 rpm

### 2.6 Characterization of yarns

The coefficient of mass variation (CVm%), imperfections, and hairiness of yarns were ascertained by Uster® Tester 6 (UT 6, Uster Technologies, Switzerland) in accordance with ASTM D1425/1425M-14(2020) standard with the testing speed of 400 m/min. Spun yarns manufactured from staple fibers contain imperfections that are subdivided into thin places, thick places and neps (Thilagavathi and Karthik, 2016). Herein, the sensitivity thresholds of thick place (+50%), thin place (−50%), and neps (+200%) per 1000 m of yarn were considered for data analysis. Yarn hairiness (H), measured by the hairiness sensor of the evenness tester based on optical principle, is the total length of protruding fibers (in cm) within the measurement field of 1 cm length of yarn.

Tensile properties of yarns were studied by Tenso Lab-4, Mesdan Strength Tester, Italy operated with a constant rate of extension principle according to EN ISO 2062:2009 with a 50 N load cell, a gauge length of 500 mm, and a crosshead speed of 500 mm/min. All the samples were conditioned before testing according to ISO 139. All the tests were carried out in the laboratory under a standard testing atmosphere at 65 ± 2% relative humidity and 25 ± 2 °C temperature.

The surface morphology of the yarns was observed with an optical microscope Euromex BV, Model NZ 1703-M, Netherlands.

Sample single jersey knit fabrics were constructed by Mesdan Lab Knitter, Italy having cylinder diameter: 3 inches, cylinder speed: 225 rpm, number of needle per inch: 12, and number of feeder: 1.

## 3 Results and discussion

### 3.1 Visual appearance of yarns

**Figure 8:**
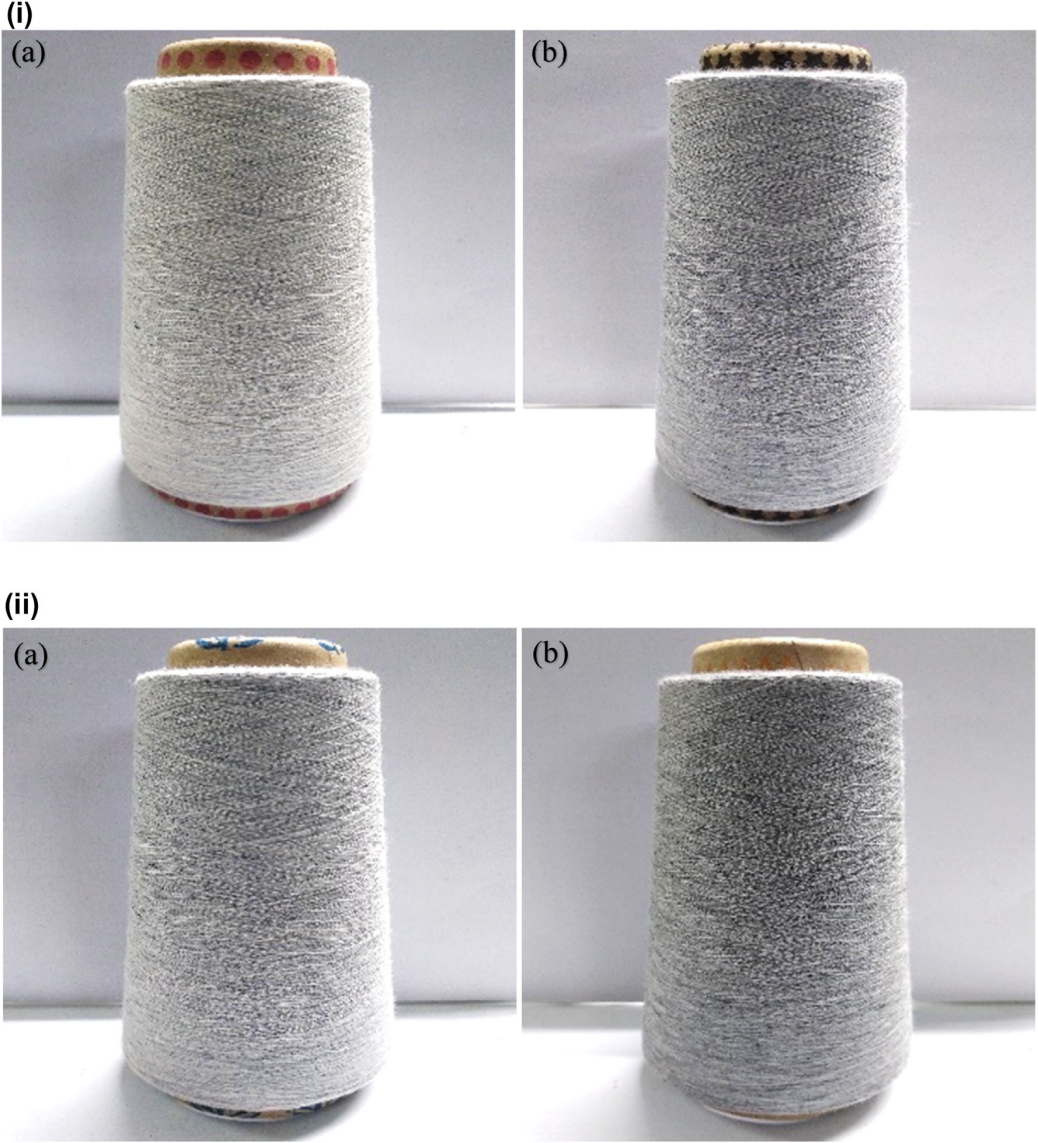
(i) Winding packages of 30 Ne ring-spun yarn produced from pre-consumer recycled cotton and virgin cotton with blend ratio (a) 10:90 and (b) 30:70. (ii) Winding packages of 30 Ne ring-spun yarn produced from post-consumer recycled cotton and virgin cotton with blend ratio (a) 10:90 and (b) 30:70.

In this study, pre- and post-consumer recycled cotton fibers were blended with virgin cotton in blow room at four different percentages i.e. 10%, 20%, 25% and 30%, and 30 Ne yarns suitable for knit top garments were produced in ring frame. Figure 8(i) and (ii) show the representative images of the yarns produced from pre-consumer and post-consumer recycled cotton with the lowest and highest blend ratios i.e. (a) 10:90 and (b) 30:70, respectively.

As seen in Figure 8(i), with the increase in pre-consumer recycled cotton derived from (50% hard waste +25% woven cut clips +25% blue knit cut clips), the darkness of yarn color (arising from blue knit cut clips) increases.

Similarly, as seen in Figure 8(ii), with the increase in post-consumer recycled cotton generated from 100% black garments waste, the darkness of yarn color increases accordingly.

Both kinds of yarns produced with partially colored pre-consumer recycled cotton and fully-colored post-consumer recycled cotton have the visual appearance of mélange yarns. The shades of mélange effect of the yarns produced here can be manipulated by varying the ratio of colored pre- and post-consumer recycled cotton fiber in yarn. The outcome of this study may be considered to be a sustainable and eco-friendly approach to producing mélange yarn using recycled fibers from colored fabric waste as an alternative to colored viscose fiber (Wang et al., 2020).

### 3.2 Structural aspects of yarns

#### 3.2.1 Unevenness of yarns

**Figure 9:**
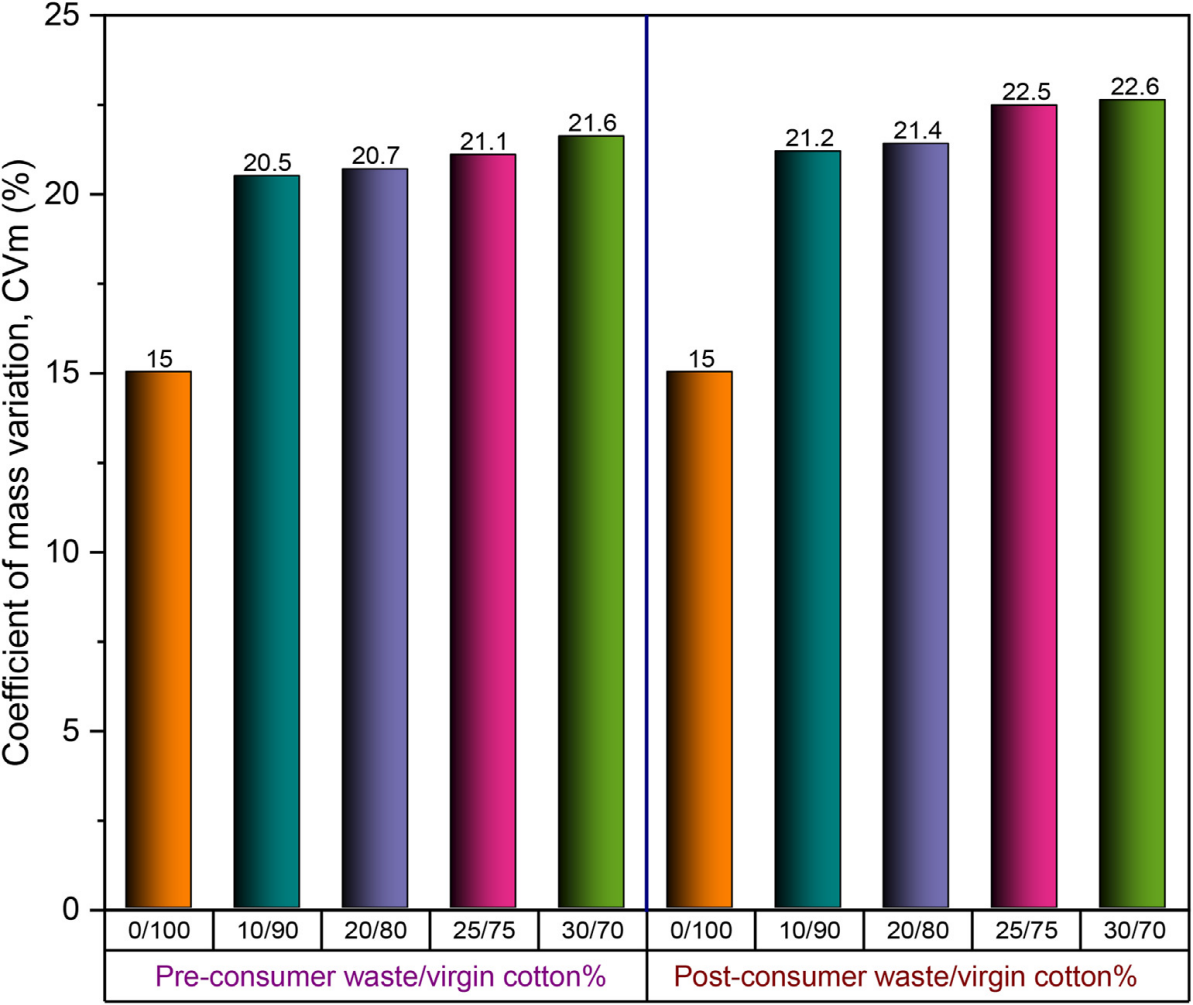
Coefficient of mass variation (CVm%) of 30 Ne ring yarn produced from pre- and post-consumer recycled cotton and virgin cotton with different blend ratios.

Unevenness of yarns is usually expressed by coefficient of mass variation (CVm%). In Figure 9, CVm% of 30 Ne ring yarns produced from pre- and post-consumer recycled cotton and virgin cotton with different blend ratios is illustrated. For comparison purposes, CVm% of yarn produced with 100% virgin cotton is also shown (by 0/100 blend ratio).

It can be observed in Figure 9 that CVm% of all yarns containing pre- and post-consumer cotton fibers is significantly higher than that of 100% cotton yarn. Besides, CVm% of yarns shows an increasing trend with the increase of recycled fiber%. The reason can be ascribed to the lower fiber length (UQL) and the presence of the remarkably high amount of short fibers in recycled fibers that were generated by vigorous opening action during the shredding operation. Compared with pre-consumer recycled fibers, the presence of short fiber% in post-consumer recycled fibers was higher due to the occurrence of fiber degradation during usage of the garments. Looking at Table 2 and Table 3, it is seen that post-consumer recycled fibers had lower UQL and higher short fiber% compared with the same of pre-consumer recycled fibers which led to a proportional increase in CVm% of yarns.

#### 3.2.2 Imperfections (thick, thin and neps) of yarns

Other than CVm%, the mass variation of yarn over the length can also be expressed by the imperfections viz. thick, thin and neps. Since imperfections of yarns are highly related to the strength values, yarns containing more imperfections poorly perform in subsequent processes, e.g. warping, sizing, weaving and knitting. Imperfections of yarn also adversely affect the visual appearance and properties of woven or knit fabric (Regar et al., 2017). The important measures that are associated with imperfections are (i) individualization of fibers, (ii) minimization of inter-fiber contact by greater fiber parallelization and (iii) control of the movement of short fibers.

**Figure 10:**
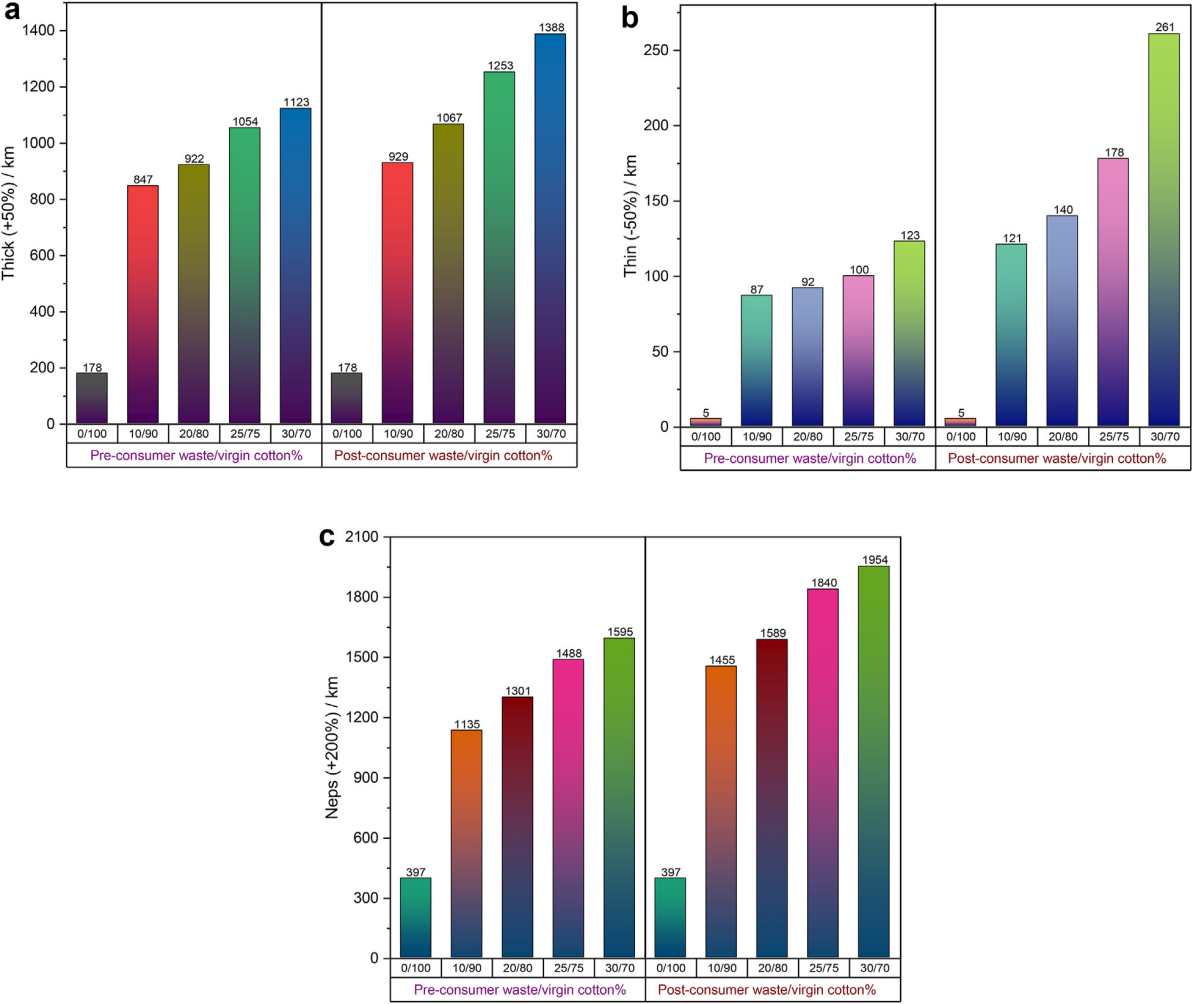
(a) Thick places of 30 Ne ring yarn produced from pre- and post-consumer recycled cotton and virgin cotton with different blend ratios. (b) Thin places of 30 Ne ring yarn produced from pre- and post-consumer recycled cotton and virgin cotton with different blend ratios. (c) Neps of 30 Ne ring yarn produced from pre- and post-consumer recycled cotton and virgin cotton with different blend ratios.

Thick (+50%) and thin (−50%) places per kilometer of the yarns are shown respectively in Figure 10(a) and (b). When compared with the same of 100% cotton yarn, an increase in pre- and post-consumer recycled fiber% in yarn has a detrimental effect on the thick and thin places which are due to the presence of an excessive amount of short fibers (Table 2 and Table 3). Thick and thin values show a gradually increasing trend with the increase of pre- and post-consumer recycled fiber content in yarn and the rate of increase is higher in the case of post-consumer blend yarns. The reason can be attributed to the lower fiber length (UQL) and higher short fiber% of post-consumer recycled fibers compared to the pre-consumer counterparts (Table 2 and Table 3). During drafting, short fibers floated between two successive rollers are dragged forward by the more rapidly moving fibers already gripped by the front rollers. This causes a relatively thick place to pass through the front rollers, leaving behind a thin place in the space between the rollers. In this way, the irregular motion of short fibers creates drafting waves in slivers that ultimately result in high thick and thin places in yarn.

Neps are consisted of chiefly small clusters of entangled fibers and are found scattered throughout the fabric surface if they are present in the yarn. They are considered as blemishes in dyed fabrics. The severity of neps may even be a reason for lot rejection after the final stage of garment production. Neps are created in ginning and blow room during opening and beating action and can be reduced by careful monitoring of carding and combing process. The complete avoidance of neps during the production of yarns from staple fibers is a fundamental technological limitation (Islam and Uddin, 2022).

Neps (+200%) of the manufactured yarns are displayed in Figure 10(c) where similar pattern like thick and thin places is visible. This can be assigned to the higher neps values of pre- and post-consumer recycled fibers (Table 2 and Table 3). Fiber neps formation is influenced far more by the shredding operation than by fiber preparation as part of the staple-fiber spinning process. Moreover, the surface characteristics of dyed fibers present in pre- and post-consumer recycled fibers are different than virgin cotton. Dyed fibers possess a different frictional property that turns the waste fabrics into short fibers and neps-like fiber clusters during mechanical shredding. Consequently, opening and drafting of these fibers become more difficult while processing a higher percentage of dyed fibers in the spinning line. Deterioration of yarn quality with the increase of color recycled fibers% of the present work is analogous with the study that the quality of melange yarn was dropping, in terms of CVm and imperfections, while increasing shade depth% of yarn by increasing dyed cotton fibers (Ray et al., 2018).

#### 3.2.3 Hairiness of yarns

**Figure 11:**
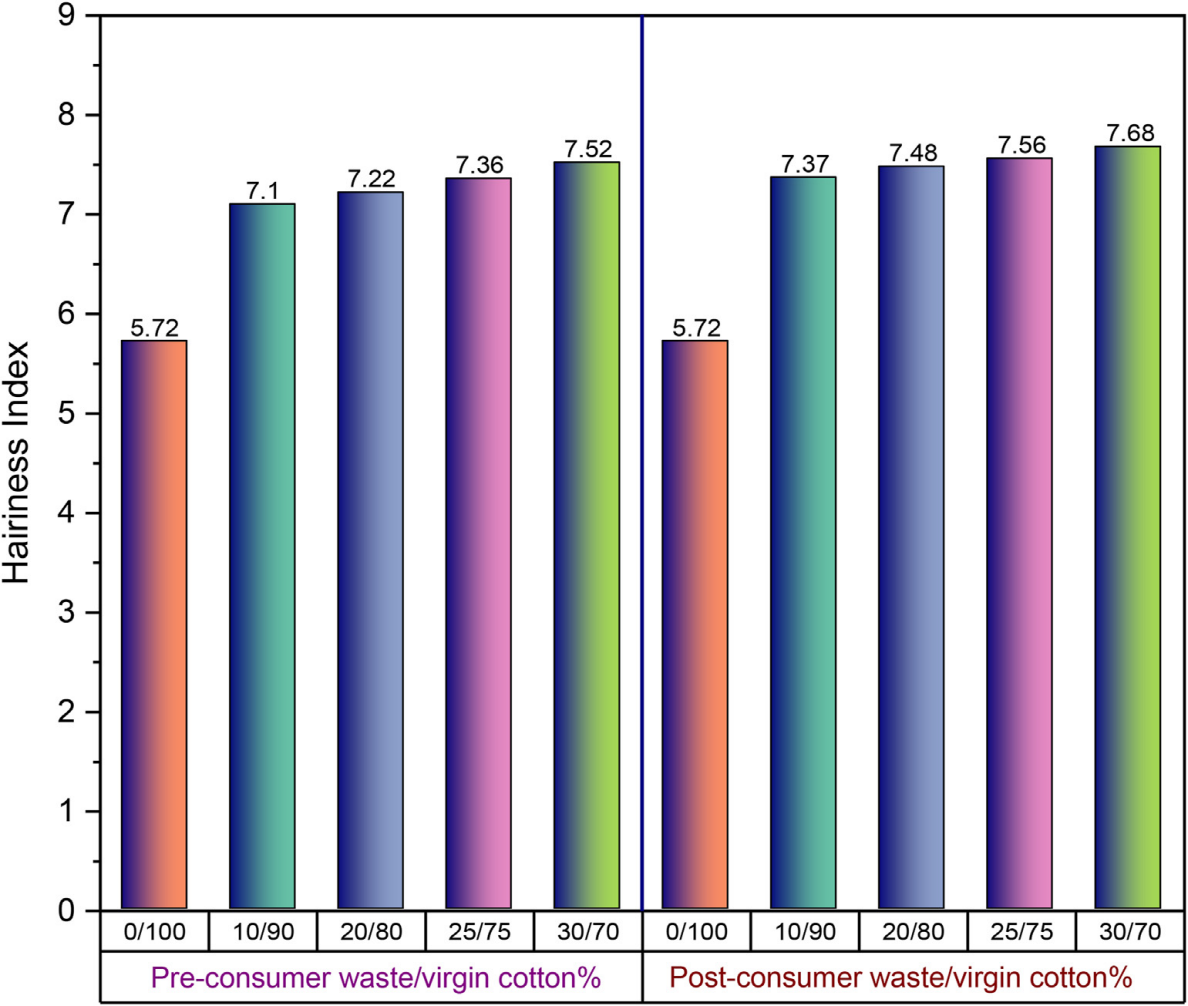
Hairiness index of 30 Ne ring yarn produced from pre- and post-consumer recycled cotton and virgin cotton with different blend ratios.

The hairiness of yarn is the result of fiber protrusion from the yarn surface. Among different properties, hairiness is also considered to be one of the significant attributes of ring yarns since the fabric performance is greatly determined by it. Depending upon the application, a certain amount of hairiness is desirable to obtain the aesthetic properties of the fabric. For instance, hairiness provides a soft feel and wicking property to the fabric, but excessive hairiness is undesirable (Haleem and Wang, 2015). Figure 11 is the graphical representation of the variation in yarn hairiness values with the increase in pre- and post-consumer recycled fiber% in yarn. Like CVm% and imperfections, similar patterns can be observed in the hairiness values of yarn. The result can be interpreted with the content of short fiber% in pre- and post-consumer recycled fibers (Table 2 and Table 3). In addition, the dyed fibers derived from pre- and post-consumer wastes are coarser (Ray et al., 2018) as found in fiber fineness values in Table 2 and Table 3. In ring spinning, shorter and coarser fibers tend to migrate toward the outer surface of the yarn body during ballooning whereas longer and finer fibers move toward the yarn core (Ray et al., 2018; Lawrence 2010). This phenomenon may be the cause of higher hairiness of yarns while increasing pre- and post-consumer color fibers% in blends.

**Figure 12:**
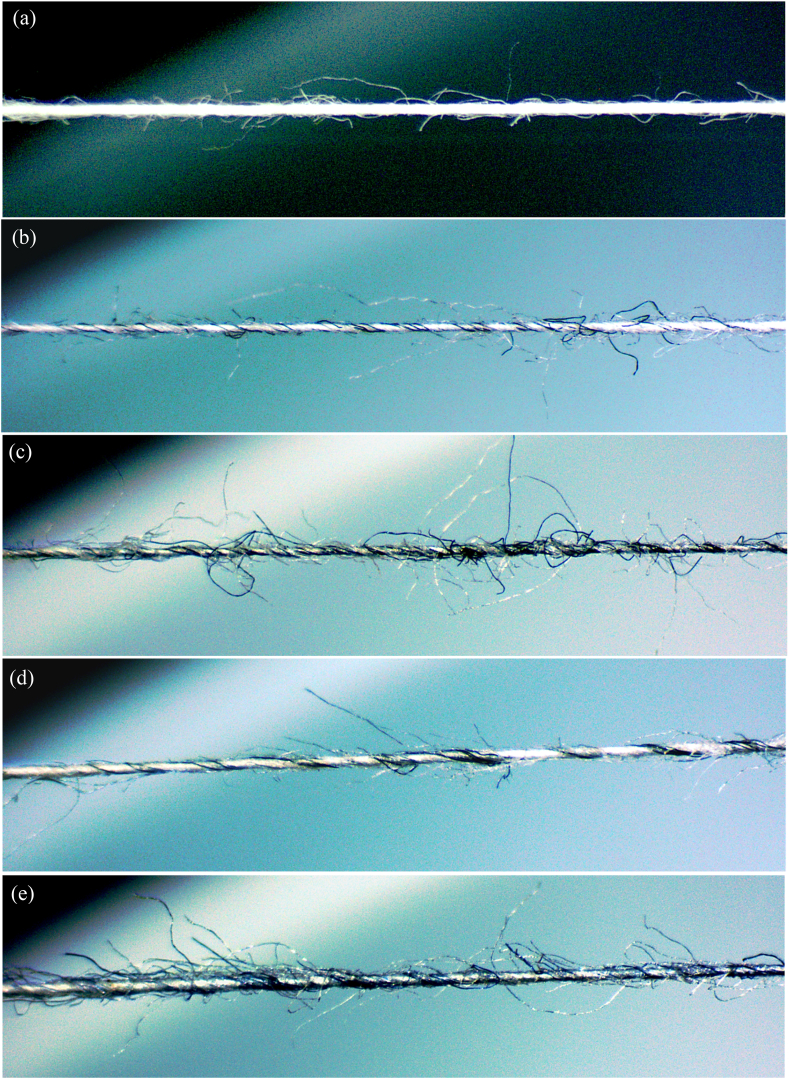
Optical microscopic images of yarns produced from (a) 100% virgin cotton, (b) pre-consumer waste 10%, (c) pre-consumer waste 30%, (d) post-consumer waste 10% and (e) post-consumer waste 30%.

Microscopic images of yarns shown in Figure 12(a–e) validate the migration of shorter and coarser dyed fibers derived from shredded pre- and post-consumer wastes at the yarn surface.

### 3.3 Tensile properties of yarns

**Figure 13:**
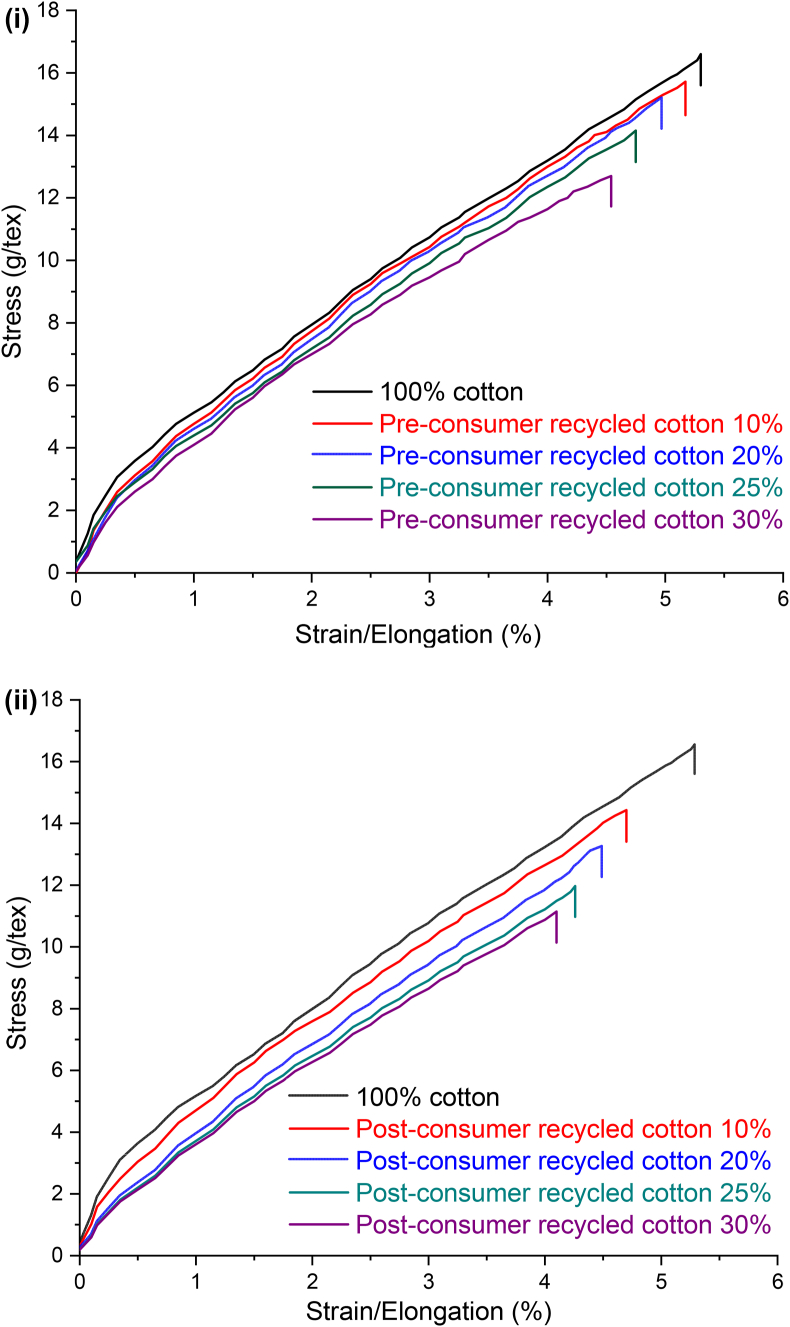
(i) Stress-strain curves of 30 Ne ring yarn produced from the blend of pre-consumer recycled cotton and virgin cotton with different blend ratios. (ii) Stress-strain curves of 30 Ne ring yarn produced from the blend of post-consumer recycled cotton and virgin cotton with different blend ratios.

**Figure 14:**
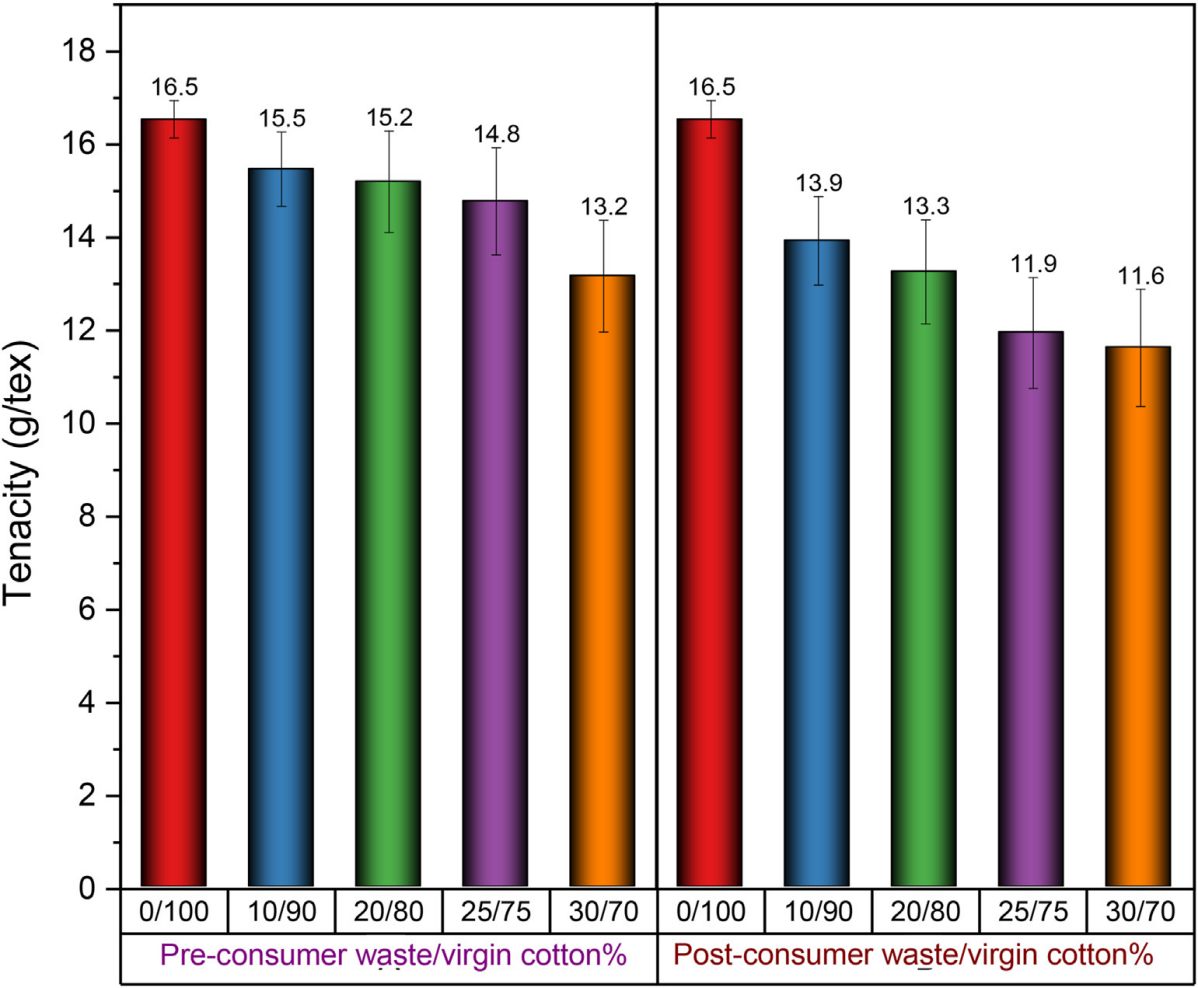
Tenacity of 30 Ne ring yarn produced from pre- and post-consumer recycled cotton and virgin cotton with different blend ratios.

**Figure 15:**
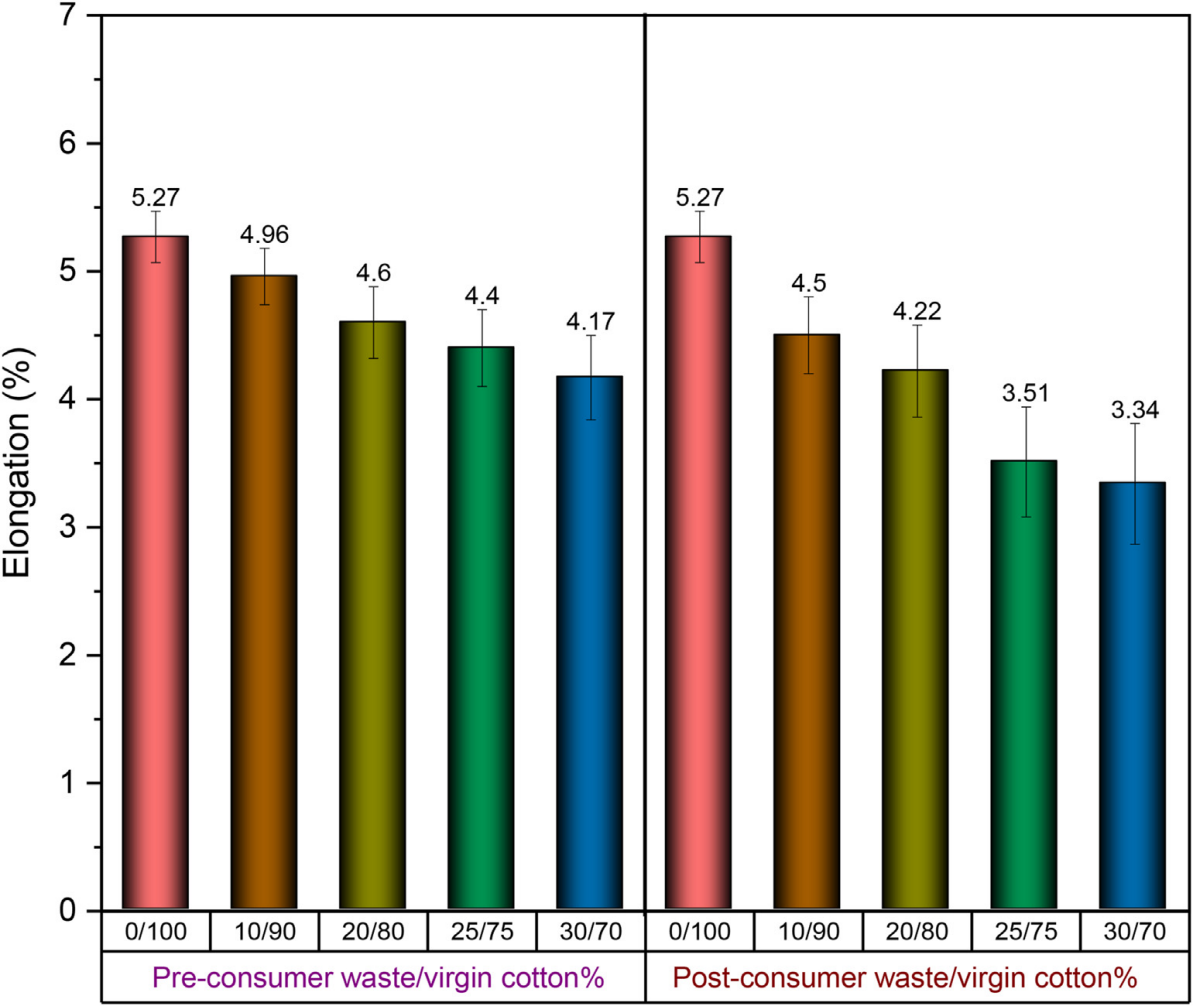
Elongation (%) of 30 Ne ring yarn produced from pre- and post-consumer recycled cotton and virgin cotton with different blend ratios.

Figure 13(i) and (ii) show the representative stress-strain curves of 30 Ne ring yarn composed of pre- and post-consumer recycled cotton blended with virgin cotton with different blend ratios. Compared to the 100% cotton yarn, all blend yarns exhibit a gradual decrease in breaking strength and elongation with the proportional increase of recycled fiber content. Tensile properties including tenacity and elongation at break, evaluated from the respective stress-strain curves, are displayed in Figure 14 and Figure 15.

In Figure 14, it is obvious that the tenacity of yarns produced from blends of virgin cotton/recycled cotton decreases proportionally with the increase of recycled fiber% in yarn and the rate of decrease is higher in the case of post-consumer recycled fibers. The strength of the yarns was found to have reflected in the efficiency and end breakage rate of the ring spinning as shown in Table 5. However, if the pattern of the strength (Figure 14) is compared with the same CVm% (Figure 9) and imperfections (Figure 10), then a concomitant deterioration of the structure and properties of blend yarns with the inclusion of recycled fibers can be found. The reason was previously interpreted as the existence of a high amount of short fiber% in the recycled cotton fibers that were generated during the shredding process. When a yarn is subjected to tensile loading, short fibers present in the yarn are more prone to slip rather than participate against the applied load, leading to lower strength value. In addition, the strength of cotton fiber deteriorates during dyeing, and the weak dyed fibers are more vulnerable to be ruptured while subjected to mechanical action during shredding (Ray et al., 2018). Moreover, fiber degradation occurs during the usage of the garments. In this work, since dyed fabrics were used as pre-consumer waste, and dyed and used fabrics were used as post-consumer waste, severe rupture of colored and weak fibers might have occurred while converting colored waste fabrics into fibers by shredding and also during the opening and cleaning operation in the spinning line (Table 2 and Table 3).

**Table 5:** Efficiency and end breakage rate during ring spinning.

Variables	Virgin cotton	Pre-consumer recycled yarn				Post-consumer recycled yarn			
		10%	20%	25%	30%	10%	20%	25%	30%
Efficiency (%)	95.2	93	92.2	91.6	91	92.8	91.9	91.3	89.5
End breakage%/100 spindle per hr	3.95	4.05	4.15	4.61	4.83	4.25	4.45	5.05	5.18

Like breaking strength, sufficient breaking elongation of yarns is required as well to enable the yarn to sustain stresses during subsequent processes during the conversion of yarn into fabric. It also determines the effective extensibility of the final product while in use (Almetwally et al., 2014). The breaking elongation of the yarns in the present study is presented in Figure 15. With the increase of recycled fiber content, the same declining pattern like strength is discernible. When a load is applied to yarn, it is distributed among the constituent fibers. The breaking elongation of yarn is related to fiber extension and the way the fibers are arranged in its body. As recycled fibers contain a great number of short fibers, they are poorly integrated with the yarn body and slipped out easily during the application of load resulting in lower elongation of yarns.

### 3.4 Statistical analysis

The correlations and significance of paired samples such as the amount of recycled fiber% of pre- and post-consumer recycled blend yarns with various yarn parameters are shown in Table 6. As seen in Table 6, a positive correlation i.e. 0.862 was found between recycled fiber% and yarn unevenness i.e., with the increase of pre-consumer recycled fiber%, yarn unevenness increased (as seen before in Figure 9). Positive correlations were also found for thick place 0.922 (Figure 10a), thin place 0.911 (Figure 10b), neps 0.954 (Figure 10c) and hairiness 0.867 (Figure 11). Negative correlation values were observed for tenacity (−0.862) and elongation at break% (−0.962) that means with the increase of pre-consumer recycled fiber%, the tenacity and elongation values decrease (Figure 14 and 15, respectively). All the relations were found to be highly significant (p = 0.000).

**Table 6:** Paired samples correlations for pre- and post-consumer recycled blend cotton yarns with various yarn properties.

Variables	Pre-Consumer Recycled Cotton Yarn		Post-Consumer Recycled Cotton Yarn	
	Correlation coefficient	Significance	Correlation coefficient	Significance
Recycled fiber% & Unevenness	0.862	0.000	0.882	0.000
Recycled fiber% & Thick	0.922	0.000	0.931	0.000
Recycled fiber% & Thin	0.911	0.000	0.946	0.000
Recycled fiber% & Neps	0.954	0.000	0.933	0.000
Recycled fiber% & Hairiness	0.867	0.000	0.843	0.000
Recycled fiber% & Tenacity	-0.862	0.000	-0.965	0.000
Recycled fiber% & Elongation	-0.962	0.000	-0.925	0.000

Similar correlation and significance were obtained for post-consumer recycled blend yarns.

### 3.5 Cost considerations for virgin cotton and recycled cotton

Cotton is a huge water-intensive crop. Production of 1 kg of cotton fiber needs about 20,000 L of water causing depletion of freshwater reserves and even drought problems in the cultivation areas. In addition, conventional cotton farming uses a lot of harmful pesticides to control pests and synthetic fertilizers to boost production. These toxic chemicals threaten human health, wildlife, water, and soil. Moreover, cotton production emits around 220 million tons of carbon dioxide yearly. Synthetic fertilizers used in cotton field release nitrous oxide which is 310 times more powerful greenhouse gas than carbon dioxide (Environmental Impact of Cotton from Growing, Farming & Consuming, 2022). Therefore, recycling cotton fiber is of great importance and its utilization in yarn manufacturing brings a great economical and environmental advantage.

The manufacturing cost of cotton yarn essentially depends on the price of the raw material and the energy cost (Wanassi et al., 2018). The detailed cost analysis is given in Table 7. The cost of recycled cotton includes the cost of waste material and shredding operation for fiber extraction. When it is blended with virgin cotton (10/90, 20/80, 25/75 and 30/70), the virgin cotton cost is added. The virgin fiber cost is the commercial cost incurred during the purchase of the fiber. The overall cost has been calculated in terms of ring frame average speed, twist multiplier (TM) of yarn, power consumption, salaries and wages, stores, administrative, interest and depreciation. The overhead cost for recycled yarn increases with the increase of recycled fiber% in yarn due to the lower speed and efficiency of ring frame, and the higher amount of pneumatic waste. However, the total cost of yarn manufacturing decreases with the increase of recycled fiber%.

**Table 7:** Cost for raw material and yarn manufacturing from virgin cotton and virgin cotton/recycled cotton blends.

Components of cost	Virgin cotton purchasing cost (US$/Kg)	100% Recycled fiber re-claiming cost (US$/Kg)	Recycled fiber: Virgin cotton (10:90) cost (US$/Kg)	Recycled fiber: Virgin cotton (20:80) cost (US$/Kg)	Recycled fiber: Virgin cotton (25:75) cost (US$/Kg)	Recycled fiber: Virgin cotton (30:70) cost (US$/Kg)
Raw material cost	2.05	0.28	1.87	1.70	1.61	1.52
Fiber extraction cost	–	0.30	0.03	0.06	0.08	0.09
Total Fiber cost	**2.05**	**0.58**	**1.90**	**1.76**	**1.68**	**1.61**
Overall overhead cost for manufacturing per kg yarn	1.10	–	1.15	1.20	1.22	1.27
Total yarn manufacturing cost per kg yarn	**3.15**	–	**3.05**	**2.96**	**2.90**	**2.88**

### 3.6 Analysis of yarn characteristics for possible applications

Mélange yarn is usually produced by blending dyed viscose fibers with virgin cotton where a unique mixed color effect is obtained. Mélange yarn offers a great variety of shades depending upon the proportion of dyed viscose fiber. Since the strength of viscose fiber is much lower than the same of cotton, the strength of melange yarn is lower than the yarn composed of 100% cotton fiber and the deterioration of strength becomes more with the increase of shade depth% i.e. ratio of colored viscose fiber%. During the production of mélange or similar-type fancy yarn, a part of yarn strength is sacrificed to attain new aesthetics along with a softer handle and good absorbance property of the fabric. However, a minimum strength of yarn needs to be ensured so that it can endure the applied stress during weaving preparatory processes (warping and sizing) and fabric production (weaving and knitting) (Regar et al., 2017).

The load placed on the yarn during knitting is lower than in weaving. In spun yarns, the strength is proportional to the level of inserted twist. High twist holds the fibers tightly, making the yarn compact and thus restricting water/perspiration to penetrate. For knitting, soft-twisted yarn is desirable to obtain fabric with a soft handle and good absorbency. Elongation of yarn is necessary for knitting to sustain bending strain during loop formation and knock over. The elongation of spun yarn is inversely proportional to the twist level.

From the application point of view, the minimum tenacity required for knit yarn is 13.5 g/tex and elongation is about 3%. For woven fabric (as weft yarn), the required tenacity is above 14.0 g/tex with at least 2% elongation. In light of the strength and elongation of yarns, some conclusive remarks may be drawn from the data illustrated in Figure 14 and 15.

(a) Ring yarn containing up to 25% pre-consumer recycled cotton is suitable for knit fabric.
(b) Ring yarn containing up to 10% post-consumer recycled cotton is suitable for knit fabric.
(c) Ring yarn containing up to 25% pre-consumer recycled cotton is suitable for woven fabric (weft yarn).
(d) None of the ring yarns containing post-consumer recycled cotton is suitable for woven fabric (weft yarn).

The yarns produced in this work were aimed at knitwear and hence lower TM (around 4) was used to obtain soft and absorbent fabric. Moderately higher twist factor i.e. 4.5 and above is generally used in the case of weaving yarn since yarns have to bear a high level of stress and friction during warping, sizing, beaming and weaving processes. By increasing TM, yarn containing post-consumer recycled fiber may also be made useable for weaving.

**Figure 16:**
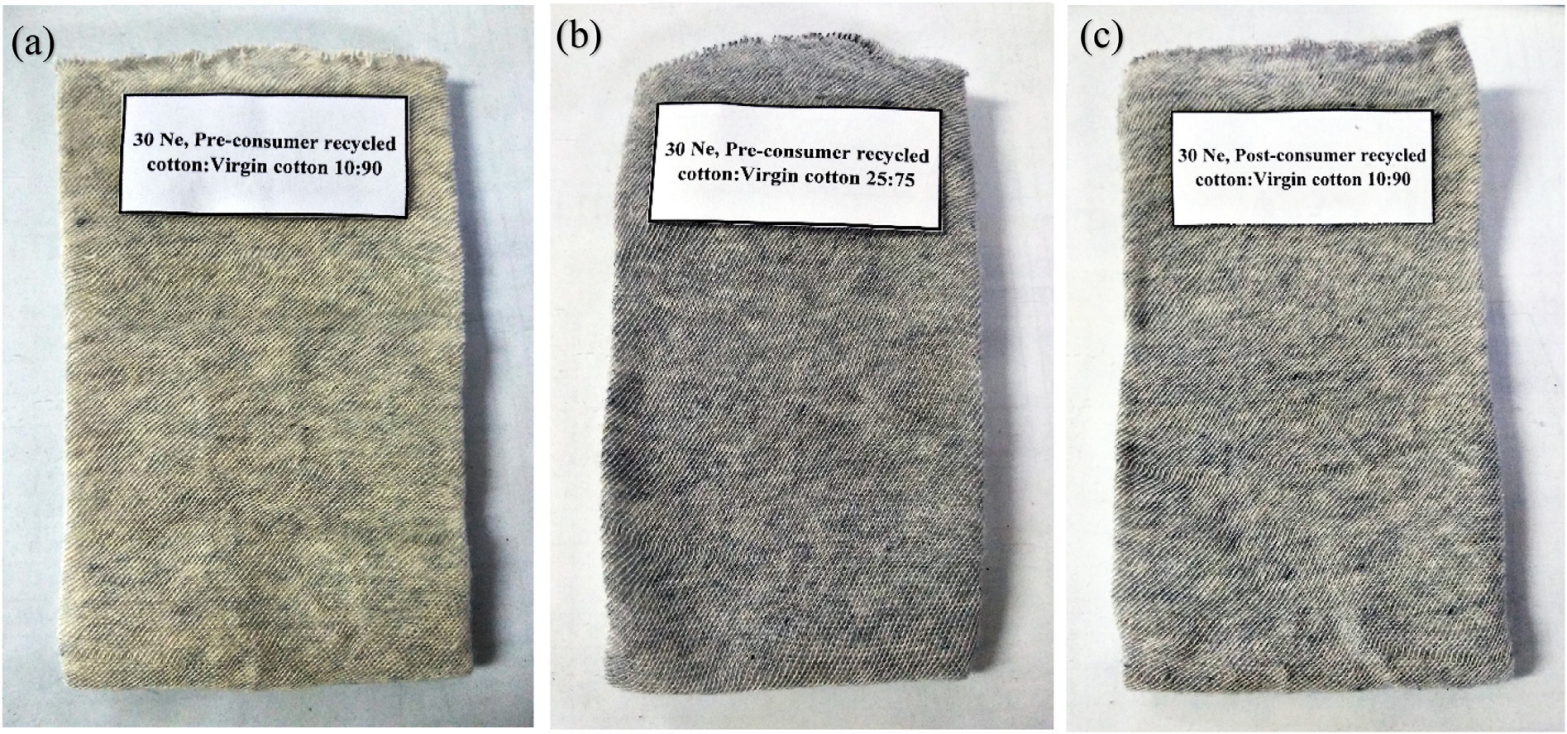
Sample knit fabrics produced from 30 Ne ring yarns containing (a) pre-consumer recycled cotton 10%, (b) pre-consumer recycled cotton 25% and (c) post-consumer recycled cotton 10%.

Finally, sample knit fabrics were produced in a lab-scale small-diameter circular knitting machine in order to check the possible production of knitwear like T-shirts and polo shirts with the produced yarns. As shown in Figure 16(a)–(c), the produced fabrics have a mélange-like appearance and their hand feel is also soft and comfortable. The abrasion- and pilling resistance along with air permeability of the fabrics produced from virgin cotton/recycled fiber blend yarns in rotor spinning were found to be decreased with the proportion of recycled fibers in yarns due to the presence of a high amount of short fibers in yarns (Awgichew et al., 2021). However, the current work focuses on the production of soft (low twist) yarns from pre- and post-consumer recycled fibers in ring spinning suitable for top knit garments. Manufacturing fabrics with detailed characterization is necessary to draw a concrete conclusion.

Mélange yarns are popular as they impart an attractive and very distinct fabric appearance. Melange yarns are mostly produced by blending dyed viscose fiber with virgin cotton during spinning. Fabric made with melange yarn does not require further dyeing and thus mélange yarn processing can save up to 50% dyeing water and reduce 50% wastewater which features apparent advantages of energy-saving, emission reduction and environmental protection (Likhon, 2020). In this study, mélange-like yarns were produced by blending colored recycled cotton fibers recovered from consumer waste with virgin cotton. Like typical mélange yarn, shades of the mélange effect can be varied by changing the ratio of colored recycled fibers in yarn.

## 4 Conclusions

The outcome of the present study can be concluded as follows:

- Recycled cotton fibers derived from shredded pre- and post-consumer textile wastes contained a high amount of short fibers and neps; yet by blending with virgin cotton, 30% waste fibers were successfully utilized in manufacturing medium count yarn (30 Ne) with soft twists in ring frame suitable to produce knit top garments.
- Compared with the properties of the yarn made with 100% virgin cotton, the blend yarns containing recycled fibers showed higher CVm and imperfections along with lower strength and elongation values with the proportional increase of recycled fiber%. The deterioration of yarn properties was higher in the case of blending post-consumer waste fiber than in pre-consumer one.
- By using and varying the colored recycled cotton fibers with virgin cotton, mélange-like fancy yarns with a wide range of color shades can be produced.
- In terms of yarn strength, blend yarn containing up to 25% pre-consumer recycled fibers was found to be suitable to produce knit- and woven fabric. Blend yarn containing up to 10% post-consumer waste was suitable for knit fabric.
- Though the properties of blend yarns were compared with the 100% cotton yarns, during producing fancy effects in yarn as mélange in the present case, the comparison of yarn quality with the virgin raw yarn seems to be impracticable. This is because, for the preparation of slub or neppy yarn, unevenness and imperfections are deliberately introduced in yarns. In the present case, fabrics having slightly higher unevenness and imperfections may be another new approach to modern consumers who are eager to accept new designs.
- Concerning the strength of yarns, lower yarn strength resulting from the addition of recycled fiber may slightly shorten the lifespan of the produced garments. A slightly shorter lifespan of garments can be mitigated by the benefit of sustainable raw material utilization in producing fancy yarn. Consumers these days are aware of the negative impacts of purchasing items that are not produced sustainably or ethically.

## Declarations

### Author contribution statement

Yeasin Arafat: Conceived and designed the experiments; Performed the experiments; Contributed reagents, materials, analysis tools or data; Wrote the paper.

Ahmed Jalal Uddin: Conceived and designed the experiments; Performed the experiments; Analyzed and interpreted the data; Contributed reagents, materials, analysis tools or data; Wrote the paper.

### Funding statement

This research did not receive any specific grant from funding agencies in the public, commercial, or not-for-profit sectors.

### Data availability statement

Data will be made available on request.

### Declaration of interest's statement

The authors declare no conflict of interest.

### Additional information

No additional information is available for this paper.
